# Clinical effects of probiotic supplementation on depressive symptom severity in adults with depressive disorders: a systematic review and meta-analysis

**DOI:** 10.3389/fnut.2026.1900466

**Published:** 2026-10-05

**Authors:** You-Sheng Zhang, Li-Shuang Zhang, Kun Zhang, Xiao-Li Liu

**Affiliations:** 1 Department of Clinical Nutrition and Metabolic Diseases, Shijiazhuang People’s Hospital, Shijiazhuang, Hebei, China; 2 Department of Endocrinology, Shijiazhuang People’s Hospital, Shijiazhuang, Hebei, China; 3 Department of Psychiatry and Psychology, The Third Hospital of Shijiazhuang, Shijiazhuang, Hebei, China

**Keywords:** depressive disorders, depressive symptom severity, meta-analysis, microbiota–gut–brain axis, probiotics, randomized controlled trials, systematic review

## Abstract

**Background:**

Probiotic supplementation has been investigated as a potential adjunctive treatment for depressive disorders, but its clinical efficacy and safety remain uncertain. This systematic review and meta-analysis aimed to evaluate the effects of probiotics on depressive symptom severity, clinical response, remission, treatment discontinuation, and adverse events in adults with clinically diagnosed depressive disorders.

**Methods:**

This systematic review and meta-analysis was conducted in accordance with PRISMA guidelines. PubMed, Embase, Web of Science, and the Cochrane Library were searched from inception to May 8, 2026. Randomized controlled trials (RCTs) evaluating orally administered probiotics against placebo or another non-probiotic control were included. Continuous outcomes were summarized as standardized mean differences (SMDs) using Hedges’ g, and dichotomous outcomes as risk ratios (RRs), with 95% confidence intervals (CIs). Random-effects models used restricted maximum likelihood estimation with Hartung–Knapp adjustment. Heterogeneity was assessed using Cochran’s Q, I^2^, and τ^2^, and robustness was examined using leave-one-out sensitivity analysis.

**Results:**

Nine RCTs involving 460 participants were included. Probiotic supplementation was associated with a modest reduction in depressive symptom severity (SMD, −0.43; 95% CI, −0.73 to −0.12; I^2^ = 46.8%; τ^2^ = 0.069). The 95% prediction interval ranged from −1.10 to 0.25. Leave-one-out estimates ranged from −0.52 to −0.38, with all 95% CIs excluding zero. No significant effect modification was detected across formulation, dose, duration, treatment context, diagnostic population, or outcome-rater subgroups. Clinical response and remission were available from one trial and remained inconclusive (RR, 0.75; 95% CI, 0.32–1.77; and RR, 0.42; 95% CI, 0.08–2.19, respectively). All-cause discontinuation did not differ clearly between groups (seven RCTs; RR, 1.15; 95% CI, 0.67–1.98). Adverse-event evidence was limited and inconsistently reported.

**Conclusion:**

Probiotic supplementation may modestly reduce depressive symptom severity, particularly as an adjunct to established treatment. However, the prediction interval included the null, and evidence regarding clinical response, remission, and safety remained inconclusive. Larger, adequately powered RCTs with standardized clinically meaningful outcomes and strain-specific reporting are required.

## 1 Introduction

Depressive disorders comprise a group of psychiatric conditions characterized by persistent disturbances in mood, cognition, somatic function, motivation, and social functioning (1). Although their diagnostic subtypes differ in duration, severity, recurrence pattern, and associated clinical features, depressive disorders share a substantial burden related to impaired quality of life, reduced occupational and social functioning, increased health-care utilization, and elevated risk of relapse (2). Current treatment strategies primarily include antidepressant pharmacotherapy, psychotherapy, or their combination. However, a considerable proportion of patients show delayed response, incomplete remission, residual symptoms, poor adherence, or adverse effects related to long-term medication use (3). Therefore, adjunctive interventions with acceptable tolerability and biologically plausible mechanisms have attracted increasing attention in the management of depressive disorders (4–6).

The microbiota–gut–brain axis provides a biologically plausible framework linking intestinal microbial activity with depressive disorders through immune-inflammatory signaling, hypothalamic–pituitary–adrenal axis regulation, vagal signaling, intestinal barrier function, microbial metabolites, and tryptophan metabolism (7–9). Microbial dysbiosis may contribute to systemic inflammation, impaired barrier integrity, altered short-chain fatty acid production, and activation of the kynurenine pathway, although findings remain heterogeneous across studies (10, 11). The International Scientific Association for Probiotics and Prebiotics defines probiotics as “live microorganisms that, when administered in adequate amounts, confer a health benefit on the host.” Probiotics investigated for potential psychological or psychiatric benefits are commonly termed psychobiotics (12). These interventions may influence microbial composition, epithelial integrity, inflammatory activity, neuroendocrine stress responses, and neuroactive metabolites. Commonly studied taxa include *Lactobacillus* and *Bifidobacterium*, although effects are likely to be strain- and formulation-specific. Recent randomized controlled trials have evaluated single-strain preparations, including *Lactiplantibacillus plantarum* PS128 and *Bifidobacterium breve* CCFM1025, as well as multistrain formulations, either alone or as adjuncts to conventional treatment in adults with depressive disorders (13–15).

Previous systematic reviews have suggested that probiotics may improve depressive symptoms; however, the clinical applicability of these findings remains uncertain because study populations were not always restricted to patients with formally established depressive disorders (16, 17). Some reviews included participants identified either by a clinical diagnosis or by elevated symptoms of depression or anxiety and allowed probiotic interventions combined with other nutritional components, whereas others evaluated major depressive disorder within broader network meta-analyses involving multiple pharmacological and microbiota-targeted interventions (18, 19). Consequently, the effects of orally administered probiotics specifically in adults with clinically diagnosed depressive disorders remain incompletely characterized. To address this gap, we conducted an updated systematic review and meta-analysis to evaluate the efficacy and safety of probiotic supplementation in adults with depressive disorders.

## 2 Methods

### 2.1 Search strategy

This systematic review and meta-analysis was conducted in accordance with the Preferred Reporting Items for Systematic Reviews and Meta-Analyses (PRISMA) guidelines (20). The review protocol was prospectively registered with PROSPERO (registration number: CRD420261468438). A comprehensive literature search was conducted in PubMed, the Cochrane Library, Web of Science, and Embase from database inception to May 8, 2026. The search syntax was adapted to the indexing systems and retrieval rules of the individual databases. The complete database-specific search strategies, including any filters or limits applied, are presented in Supplementary Table 1. The reference lists of eligible studies and relevant systematic reviews were also manually screened to identify additional potentially eligible reports. All retrieved records were imported into EndNote 21 (Clarivate, Philadelphia, PA, USA). Duplicate records were identified using EndNote’s built-in duplicate-detection function and subsequently verified and removed manually by two reviewers. No automation tools were used to exclude records before title and abstract screening. Two reviewers independently screened the titles and abstracts of the remaining records. Potentially eligible reports were retrieved in full text and independently assessed against the predefined eligibility criteria. Reasons for full-text exclusion were documented. Reports relating to the same trial were linked and treated as a single study. Disagreements at any stage were resolved through discussion, with adjudication by a third reviewer when consensus could not be reached.

### 2.2 Inclusion and exclusion criteria

Studies were selected according to predefined population, intervention, comparator, outcome, and study design (PICOS) criteria.

Eligible participants were adults aged 18 years or older with a clinically established depressive disorder. Eligible diagnoses included major depressive disorder, a current depressive episode, recurrent depressive disorder, mixed depressive-anxiety disorder, or another clinically defined depressive disorder diagnosed according to the Diagnostic and Statistical Manual of Mental Disorders, the International Classification of Diseases, or a clearly documented clinical psychiatric assessment. Studies that enrolled participants solely on the basis of elevated depressive symptom scores, without a clinical diagnosis of a depressive disorder, were excluded. Comorbid irritable bowel syndrome was neither required for inclusion nor used as an exclusion criterion.

Eligible interventions consisted of orally administered probiotics containing one or more live microbial strains or species, administered either as monotherapy or as an adjunct to conventional psychiatric treatment. Eligible comparators included placebo, usual care, antidepressant treatment alone, or another non-probiotic control. In adjunctive-treatment trials, the probiotic and control groups were required to receive comparable background treatment so that the additional effect of probiotic supplementation could be evaluated.

The primary outcome was depressive symptom severity at the end of the intervention, assessed using a validated depression scale, including the Hamilton Depression Rating Scale, Montgomery–Åsberg Depression Rating Scale, Beck Depression Inventory, Beck Depression Inventory-II, Patient Health Questionnaire-9, or another validated instrument. Secondary outcomes included clinical response, remission, all-cause discontinuation, discontinuation due to adverse events, overall adverse events, gastrointestinal adverse events, and serious adverse events. Clinical response and remission were accepted according to the definitions used by the original investigators.

Only randomized controlled trials reported as full-text articles were eligible. Studies were excluded if they met any of the following criteria: participants were healthy individuals, had stress symptoms alone, or lacked a clearly defined diagnosis of a depressive disorder; participants were younger than 18 years and adult data could not be extracted separately; the intervention consisted exclusively of prebiotics, synbiotics, fermented foods, herbal products, vitamins, or other nutritional supplements without a separately assessable probiotic component; probiotics were combined with another intervention in a manner that prevented isolation of their independent effect; depressive symptom severity was not assessed using a validated scale or the relevant outcome data were unavailable; or the report was non-randomized, observational, a case report, case series, review, protocol, conference abstract, animal study, or *in vitro* study.

For multiple reports arising from the same trial, reports were linked at the study level, and the most complete and relevant source was used for each outcome. Related reports were used, when necessary, to supplement missing intervention or outcome information without counting the same participants more than once.

### 2.3 Data extraction

Data extraction was performed independently by two reviewers using a predefined standardized form. The extracted information included the first author, publication year, study design, study population, diagnostic condition, probiotic intervention, comparator, treatment duration, concomitant treatment, depression assessment outcomes, secondary outcomes, and adverse events. For multi-arm trials, only data from the eligible probiotic group and the corresponding non-probiotic control group were extracted. For studies evaluating probiotics as adjunctive therapy, data were extracted only when the control group received the same background treatment without probiotic supplementation, thereby allowing estimation of the additional effect of probiotics. The primary outcome was depressive symptom severity after intervention, assessed using validated depression rating scales. When multiple depression scales were reported in the same study, the scale prespecified as the primary outcome in the original trial was preferentially extracted. If the primary scale was not clearly specified, the most clinically relevant and commonly used depression scale was selected for quantitative synthesis. Secondary outcomes included anxiety symptoms, cognitive function, biological markers, gut microbiota-related outcomes, treatment acceptability, tolerability, and adverse events. Any disagreement between the two reviewers was resolved through discussion, and unresolved discrepancies were adjudicated by a third reviewer.

### 2.4 Quality assessment

The risk of bias in the included randomized controlled trials was independently assessed by two reviewers using the Cochrane Risk of Bias tool, version 1 (RoB 1) (21). The following domains were evaluated: random sequence generation, allocation concealment, blinding of participants and personnel, blinding of outcome assessment, incomplete outcome data, selective outcome reporting, and other potential sources of bias. Each domain was classified as having a low, high, or unclear risk of bias. A low-risk judgment was assigned when the reported methods were adequate and unlikely to materially affect the outcome. A high-risk judgment was assigned when methodological limitations were considered likely to affect the validity of the findings. An unclear-risk judgment was assigned when the available information was insufficient to support a definitive assessment. Outcome-specific judgments were made where appropriate. The two reviewers completed the assessments independently and subsequently compared their judgments. Disagreements were resolved through discussion, with adjudication by a third reviewer when consensus could not be reached.

### 2.5 Statistical analyses

All meta-analyses were performed using Stata version 18.0 (StataCorp LLC, College Station, TX, USA). Continuous outcomes assessed using different depression scales were summarized as standardized mean differences (SMDs) with 95% confidence intervals (CIs). Hedges’ g was used to account for small-sample bias, and effect directions were harmonized so that negative values favored probiotic supplementation. Dichotomous outcomes were summarized as risk ratios (RRs) with 95% CIs. For clinical response and remission, RR values greater than 1 favored probiotics, whereas RR values below 1 favored probiotics for discontinuation and adverse-event outcomes. When at least two studies provided sufficiently comparable data, effect estimates were pooled using a random-effects model. Between-study variance was estimated using the restricted maximum likelihood method, and the Hartung–Knapp adjustment was applied to the 95% CIs of pooled estimates. Heterogeneity was evaluated using Cochran’s Q test, the I^2^ statistic, and τ^2^. Ninety-five percent prediction intervals were calculated for random-effects analyses. Outcomes reported by a single study were presented as single-study estimates without heterogeneity assessment. A continuity correction was applied to binary comparisons containing a zero cell. Outcomes were not pooled when outcome definitions were insufficiently comparable, arm-specific data were incomplete, or no events occurred in either group. Exploratory subgroup analyses of depressive symptom severity were conducted according to probiotic formulation, daily dose, intervention duration, treatment context, diagnostic population, and outcome rater. Subgroup differences were assessed using interaction tests. Leave-one-out sensitivity analysis was performed by sequentially excluding each study and recalculating the pooled estimate. Because only nine studies contributed to the primary outcome, funnel-plot asymmetry and regression-based tests for small-study effects were not used for inferential conclusions.

### 2.6 Certainty of evidence

The certainty of evidence for each outcome was assessed using the Grading of Recommendations Assessment, Development and Evaluation (GRADE) framework. Evidence from randomized controlled trials was initially rated as high certainty and was downgraded, when appropriate, for risk of bias, inconsistency, indirectness, imprecision, or publication bias. The final certainty was classified as high, moderate, low, or very low. Disagreements were resolved through discussion between the reviewers.

## 3 Results

### 3.1 Search results and study selection

A total of 704 records were identified through database and register searches, including 665 records from electronic databases and 39 records from registers. Before screening, 563 records were removed, including 265 duplicate records identified using EndNote and subsequently verified manually, and 298 records removed for other prespecified reasons. The remaining 141 records were screened by title and abstract, of which 109 were excluded because they did not meet the eligibility criteria. Thirty-two reports were sought for full-text retrieval; two could not be retrieved, and 30 full-text reports were assessed for eligibility. Of these, 21 reports were excluded because they were review articles (*n* = 7), sequential publications of previously identified trials (*n* = 3), reports with insufficient data for quantitative synthesis (*n* = 6), or clinical trials without a control group (*n* = 5). Ultimately, nine randomized controlled trials met the predefined eligibility criteria and were included in the systematic review and meta-analysis (13, 14, 22–28) (Figure 1).

**FIGURE 1:**
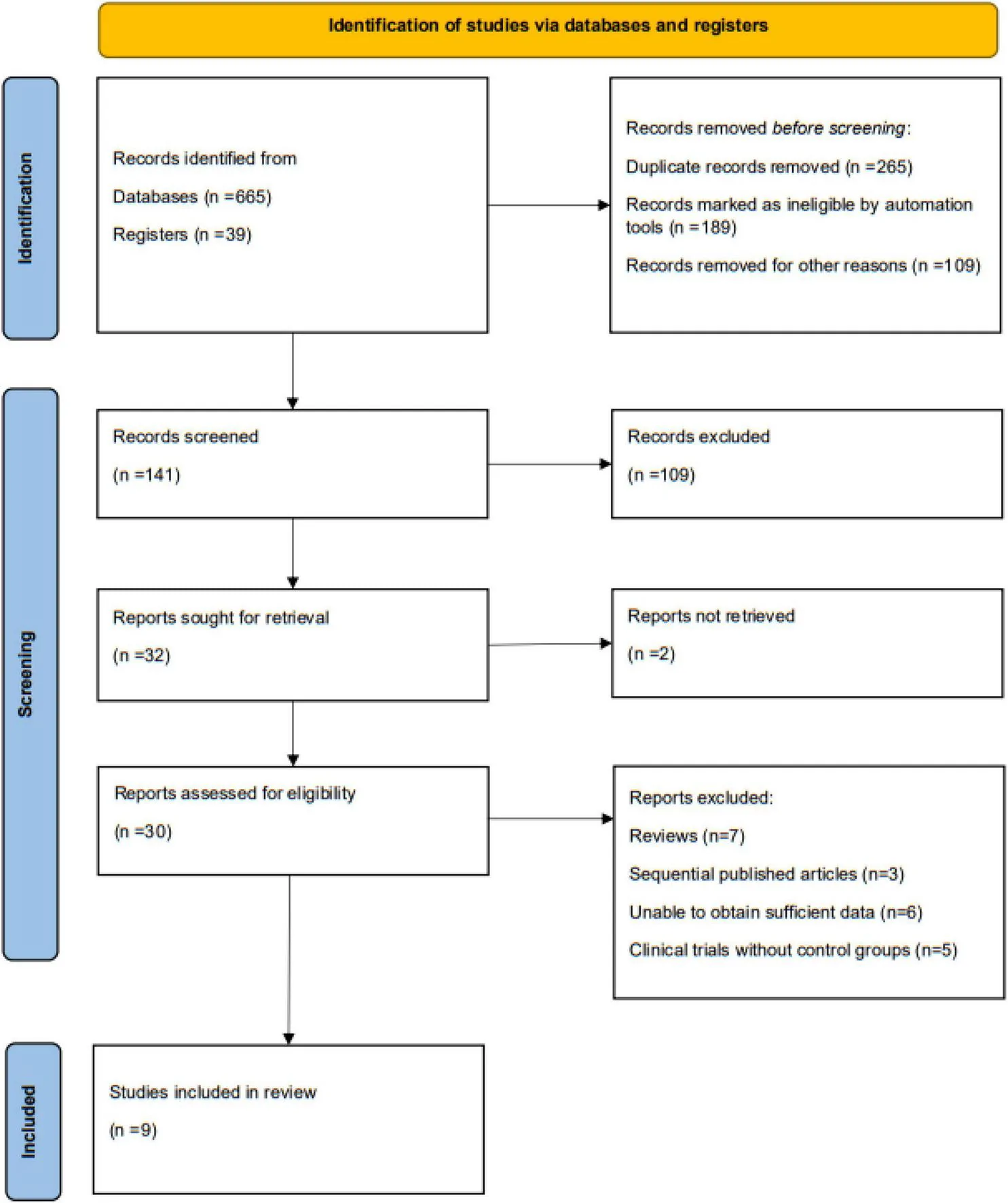
Preferred Reporting Items for Systematic Reviews and Meta-Analyses (PRISMA) flow diagram showing the literature search, screening, eligibility assessment, and final inclusion of randomized controlled trials in the meta-analysis.

### 3.2 Characteristics of the included studies

Nine randomized controlled trials were included. The studies were conducted in Iran, Taiwan, Poland, the United Kingdom, China, and Switzerland. Seven trials enrolled participants with major depressive disorder, whereas two included broader or heterogeneous depressive-disorder populations. Diagnostic criteria included the DSM-IV, DSM-IV-TR, DSM-5, ICD-10, and ICD-11; one study reported a clinical diagnosis without fully specifying the formal diagnostic framework.

Three trials evaluated single-strain probiotics, and six evaluated multistrain formulations containing between two and 14 strains. Total daily probiotic doses ranged from 3 × 10^9^ to 9 × 10^11^ colony-forming units, and intervention duration ranged from 4 weeks to 60 days. Seven trials administered probiotics as an adjunct to antidepressant treatment or inpatient treatment as usual, whereas the treatment context was mixed or not uniform in two trials.

The depression measures entered into the meta-analysis included the Hamilton Depression Rating Scale, Montgomery–Åsberg Depression Rating Scale, Beck Depression Inventory, and Beck Depression Inventory-II. Assessment was conducted at 4 weeks, 31 days, 60 days, or 8 weeks. Two trials reported study-specific definitions of clinical response; however, extractable arm-level response data were available from only one trial, which also reported remission using the Montgomery–Åsberg Depression Rating Scale. The characteristics of the included studies are presented in Table 1. Detailed probiotic formulations, doses, treatment durations, and comparators are provided in Supplementary Table 2, whereas study-specific diagnostic criteria, depression outcome definitions, and assessment time points are summarized in Supplementary Table 3.

**TABLE 1:** Study, participant, intervention, comparator, and outcome characteristics.

Part A. Study and participant characteristics											
Study		Country	Study design		Probiotic group sample size		Control group sample size		Depressive disorder		Diagnostic criteria
Elahinejad et al. (12)		Iran	Parallel-group, randomized, double-blind, placebo-controlled trial		25 randomized; 23 analyzed		25 randomized; 21 analyzed		First-episode major depressive disorder		DSM-IV-TR
Lin et al. (22)		Taiwan	Randomized, double-blind, placebo-controlled trial		16 randomized and analyzed		16 randomized and analyzed		Major depressive disorder		DSM-5
Gawlik-Kotelnicka et al. (23)		Poland	Single-center, prospective, parallel-group, randomized, double-blind, placebo-controlled trial stratified by metabolic syndrome		Arm-specific randomized N not reported; 51 analyzed		Arm-specific randomized N not reported; 44 analyzed; 43 included in the final MADRS analysis		Depressive episode, recurrent depressive disorder, or mixed depressive and anxiety disorder		ICD-11 codes 6A70, 6A71, and 6A73
Nikolova et al. (23)		United Kingdom	Single-center, parallel-group, double-blind, placebo-controlled pilot randomized clinical trial		24 randomized and included in the intention-to-treat analysis		25 randomized and included in the intention-to-treat analysis		Major depressive disorder with incomplete response to antidepressant treatment		DSM-5
Tian et al. (25)		China	Parallel-group, randomized, double-blind, placebo-controlled clinical trial		25 randomized; 20 analyzed		26 randomized; 25 analyzed		Major depressive disorder		Clinical diagnosis; HDRS-24 score > 14 was the intended eligibility criterion
Schaub et al. (14)		Switzerland	Single-center, parallel-group, randomized, double-blind, placebo-controlled add-on trial		30 randomized; 21 completed; 19 included in the modified intention-to-treat analysis		30 randomized; 26 completed; 24 included in the modified intention-to-treat analysis		Current depressive episode across affective-disorder diagnoses		ICD-10 categories F31.3–F34
Rudzki et al. (26)		Poland	Parallel-group, randomized, double-blind, placebo-controlled adjunctive trial		40 randomized; 30 analyzed		39 randomized; 30 analyzed		Major depressive disorder		DSM-IV
Kazemi et al. (27)		Iran	Three-arm, randomized, double-blind, placebo-controlled trial		38 randomized; 28 analyzed		36 randomized; 26 analyzed in the primary report; 25 included in the meta-analysis dataset		Mild-to-moderate major depressive disorder with melancholic features		DSM-IV-based clinical diagnosis
Akkasheh et al. (28)		Iran	Parallel-group, randomized, double-blind, placebo-controlled trial		20 randomized and analyzed		20 randomized and analyzed		Major depressive disorder		DSM-IV
**Part B. Intervention, comparator, and outcome characteristics**											
**Study**	**Probiotic species and strain(s)**	**Single-/multistrain**	**Total CFU/day**	**Duration**	**Treatment context**	**Background antidepressant treatment**	**Comparator and placebo**	**Depression scale used in meta-analysis**	**Assessment time**	**Clinical response definition**	**Outcome entered into meta-analysis**
Elahinejad et al. (13)	*Lactobacillus helveticus* R0052 and *Bifidobacterium longum* R0175	Multistrain (2 strains)	6 × 10^9^ CFU/day	8 weeks	Adjunctive; fluoxetine initiated concurrently	Fluoxetine 10 mg every 2 days for the first 10 days, followed by 20 mg/day	Starch placebo with identical appearance and packaging	HDRS-24	Week 8	Not assessed/reported	Week-8 HDRS-24: median 7 (IQR 4–10) versus 11 (IQR 8–19); SMD -0.86
Lin et al. (22)	*Lactiplantibacillus plantarum* PS128; DSM 28632	Single strain	6 × 10^10^ CFU/day	8 weeks	Adjunctive to stable pharmacotherapy	Antidepressant and other psychotropic treatment stable for at least 1 month; individual agents and doses not specified	Microcrystalline-cellulose placebo in visually identical capsules	HAMD-17	Week 8	Not assessed/reported	SMD +0.36 (95% CI −0.33 to +1.06)
Gawlik-Kotelnicka et al. (23)	*Lactobacillus helveticus* Rosell-52/R0052 and *Bifidobacterium longum* Rosell-175/R0175	Multistrain (2 strains)	3 × 10^9^ CFU/day	60 days	Adjunctive or stand-alone treatment	Antidepressants used by 36/51 participants in the probiotic group and 30/44 in the placebo group; some participants also received antipsychotics	Excipient-only placebo in matched capsules with double-blind coding	MADRS	Day 60	Response: ≥50% reduction from baseline; remission: MADRS score < 5	Day-60 MADRS: 16.1 ± 6.4 (*n* = 51) versus 15.9 ± 7.8 (*n* = 43); SMD +0.03; clinical response and remission event data
Nikolova et al. (24)	Bio-Kult advanced: *Bacillus subtilis* PXN 21; *Bifidobacterium bifidum* PXN 23; *B. breve* PXN 25; *B. infantis* PXN 27; *B. longum* PXN 30; *Lactobacillus acidophilus* PXN 35; *L. delbrueckii* subsp. bulgaricus PXN 39; *L. casei* PXN 37; *L. helveticus* PXN 45; *L. plantarum* PXN 47; *L. rhamnosus* PXN 54; *L. salivarius* PXN 57; *Lactococcus lactis* subsp. lactis PXN 63; and *Streptococcus thermophilus* PXN 66	Multistrain (14 strains)	8 × 10^9^ CFU/day	8 weeks	Adjunctive to stable antidepressant therapy	Stable approved antidepressant treatment for at least 6 weeks; 21/24 probiotic and 24/25 placebo participants received an SSRI	Microcrystalline-cellulose placebo in matched hydroxypropyl-methylcellulose capsules	HAMD-17	Week 8	Not assessed/reported	Week-8 HAMD-17: 8.83 ± 4.28 (*n* = 24) versus 11.09 ± 3.22 (*n* = 25); SMD −0.59
Tian et al. (25)	*Bifidobacterium breve* CCFM1025	Single strain	1 × 10^10^ CFU/day	4 weeks	Mixed treatment context; most participants received antidepressants	Antidepressants used by 18/20 participants in the probiotic group and 22/25 in the placebo group; agents and regimens varied	Maltodextrin placebo supplied as matched sachets under blinded coding	HDRS-24	Week 4	Not assessed/reported	Week-4 HDRS-24: 8.90 ± 6.40 versus 13.92 ± 8.09; SMD −0.67
Schaub et al. (14)	Vivomixx: *Streptococcus thermophilus*; *Bifidobacterium breve*; *B. longum/B. lactis*; *B. infantis/B. lactis*; *Lactobacillus acidophilus*; *L. plantarum*; *L. paracasei*; and *L. delbrueckii* subsp. bulgaricus/*L. helveticus*; strains NCIMB 30438, 30441, 30435, 30436, 30442, 30437, 30439, and 30440	Multistrain (8 strains)	9 × 10^11^ CFU/day	31 days	Adjunctive to inpatient treatment as usual	Psychotropic medications and standardized inpatient care provided as clinically indicated	Maltose placebo identical in color, shape, size, packaging, smell, and taste	HAMD	End of intervention	>57% reduction from baseline	SMD −0.19 (95% CI −0.79 to +0.42)
Rudzki et al. (26)	*Lactiplantibacillus plantarum* 299v/DSM 9843	Single strain	2 × 10^10^ CFU/day	8 weeks	Adjunctive to selective serotonin reuptake inhibitor therapy	SSRI therapy; 9/30 probiotic and 7/30 placebo participants were receiving treatment before enrollment; remaining participants initiated treatment at enrollment	Crystalline-cellulose placebo in matched capsules under double-blind coding	HAMD-17	Week 8	Not assessed/reported	SMD −0.67 (95% CI −1.19 to −0.15)
Kazemi et al. (27)	*Lactobacillus helveticus* R0052/CNCM I-1722 and *Bifidobacterium longum* R0175/CNCM I-3470	Multistrain (2 strains)	≥1 × 10^10^ CFU/day	8 weeks	Adjunctive to ongoing antidepressant therapy	Existing treatment with fluoxetine, citalopram, amitriptyline, or sertraline was maintained	Placebo containing xylitol, maltodextrin, malic acid, and plum flavor with the same appearance and taste	BDI-II	Week 8	Not assessed/reported	Week-8 BDI-II: 9.8 ± 8.0 (*n* = 28) versus 15.9 ± 12.7 (*n* = 25); SMD −0.57; prebiotic arm excluded
Akkasheh et al. (28)	*Lactobacillus acidophilus*, *Lactobacillus casei*, and *Bifidobacterium bifidum*; strain identifiers not reported	Multistrain (3 species)	6 × 10^9^ CFU/day	8 weeks	Adjunctive to citalopram	Citalopram maintained during the intervention	Starch placebo in indistinguishable capsules	BDI	Week 8	Not assessed/reported	Change in BDI: −5.7 ± 6.4 versus −1.5 ± 4.8; SMD −0.73

BDI, Beck Depression Inventory; CFU, colony-forming units; CI, confidence interval; HAMD/HDRS, Hamilton Depression Rating Scale; IQR, interquartile range; MADRS, Montgomery–Åsberg Depression Rating Scale; SMD, standardized mean difference; SSRI, selective serotonin reuptake inhibitor.

### 3.3 Risk-of-bias assessment

All nine trials were rated as having a low risk of bias for random-sequence generation and allocation concealment. One trial was rated as having a high risk of performance bias, two were rated as having a high risk of detection bias, two were rated as having a high risk of attrition bias, and one was rated as having a high risk of selective reporting. No trial was rated as having a high risk of other bias. The domain-level risk-of-bias judgments are shown in Figure 2.

**FIGURE 2:**
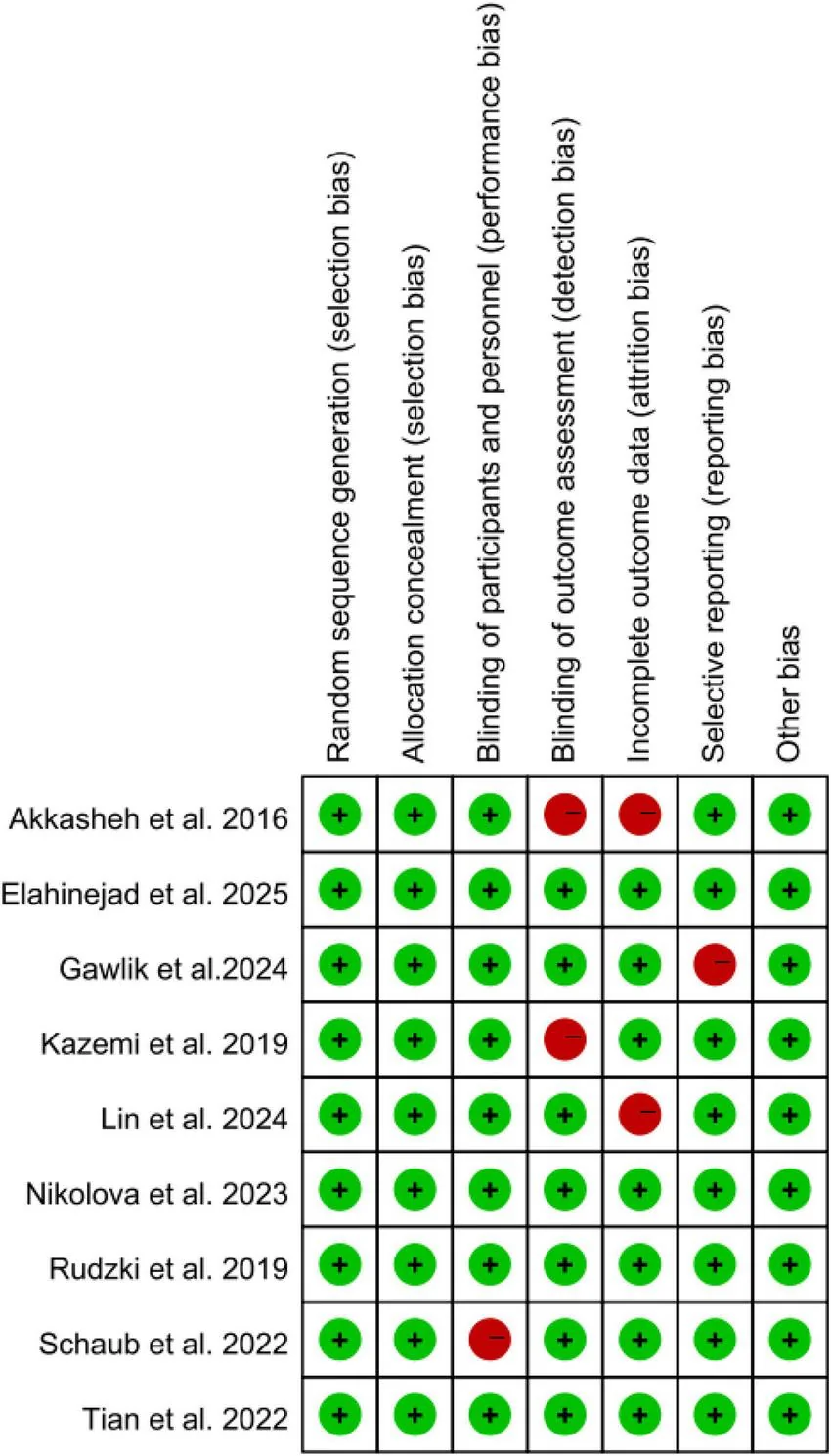
Risk-of-bias assessment of the included randomized controlled trials using the Cochrane risk-of-bias tool.

### 3.4 Depressive symptom severity

Nine trials involving 460 participants were included in the analysis of depressive symptom severity. Using a restricted maximum likelihood random-effects model with the Hartung–Knapp adjustment, the pooled standardized mean difference was −0.43 (95% CI, −0.73 to −0.12). The estimated between-study heterogeneity was I^2^ = 46.8%, with *Q*-test *P* = 0.059 and τ^2^ = 0.069. The 95% prediction interval ranged from −1.10 to 0.25 (Figure 3 and Table 2). Study-specific outcome selection, analysis populations, data sources, and data-processing procedures are detailed in Supplementary Table 4.

**FIGURE 3:**
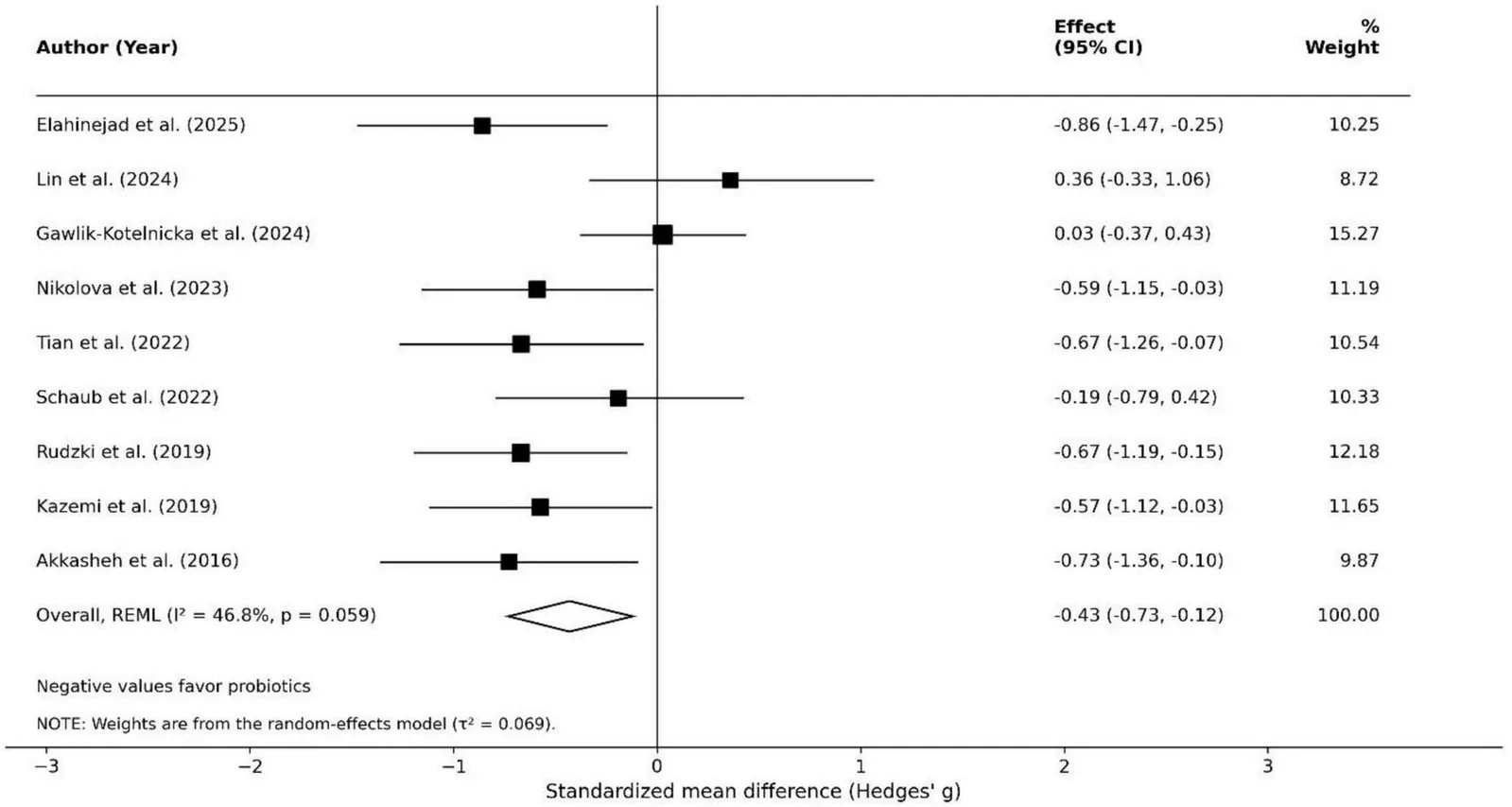
Forest plot of the effect of probiotic supplementation on depressive symptom severity in adults with depressive disorders.

**TABLE 2:** Summary of meta-analysis results.

Outcome	No. of studies	Total sample size	Effect measure	Statistical model	Pooled effect	95% CI	I^2^ (%)	*Q*-test *P*-value	τ ^2^	95% prediction interval	Direction and interpretation
Depressive symptom severity	9	460	SMD (Hedges’ g)	REML random-effects model with Hartung–Knapp adjustment	−0.43	−0.73 to −0.12	46.8	0.059	0.069	−1.10 to 0.25	Negative SMD values favor probiotics; the prediction interval includes the null.
Clinical response	1	94	Risk ratio	Single-study estimate	0.75	0.32–1.77	Not applicable	Not applicable	Not applicable	Not applicable	RR > 1 favors probiotics; the confidence interval includes substantial benefit and harm.
Remission	1	94	Risk ratio	Single-study estimate	0.42	0.08–2.19	Not applicable	Not applicable	Not applicable	Not applicable	RR > 1 favors probiotics; only six remission events were observed.
All-cause discontinuation	7	403	Risk ratio	REML random-effects model on the log-RR scale with Hartung–Knapp adjustment	1.15	0.67–1.98	1.9	0.41	0	0.67 to 1.98	RR < 1 favors probiotics; no clear between-group difference. Lin lacked extractable withdrawal counts, and Gawlik lacked randomized allocation by arm.
Discontinuation due to adverse events	1	50	Risk ratio	Single-study estimate with continuity correction	0.33	0.01–7.81	Not applicable	Not applicable	Not applicable	Not applicable	One event was reported; the estimate is uninformative.
Overall adverse events	5	368	Risk ratio	Not pooled because event definitions or arm-specific counts were incomplete	Not estimable	Not estimable	Not applicable	Not applicable	Not applicable	Not applicable	Five reports provided safety information, but a common event definition and complete arm-level counts were unavailable.
Gastrointestinal adverse events	1	60	Risk ratio	Single-study estimate	0.5	0.05–5.22	Not applicable	Not applicable	Not applicable	Not applicable	RR < 1 favors probiotics, but only three events were observed.
Serious adverse events	4	294	Risk ratio	Not estimable because no events were observed in reports with explicit statements	0 events	Not estimable	Not applicable	Not applicable	Not applicable	Not applicable	No observed events in 294 participants do not exclude rare serious harms.

CI, confidence interval; REML, restricted maximum likelihood; RR, risk ratio; SMD, standardized mean difference. Negative SMD values favor probiotics. For clinical response and remission, RR > 1 favors probiotics; for discontinuation and adverse-event outcomes, RR < 1 favors probiotics.

### 3.5 Clinical response and remission

Extractable arm-level data for clinical response and remission were available from one trial involving 94 participants. Clinical response was defined as a reduction of at least 50% from baseline in the Montgomery–Åsberg Depression Rating Scale score, with an RR of 0.75 (95% CI, 0.32–1.77). Remission was defined as a Montgomery–Åsberg Depression Rating Scale score below 5, with an RR of 0.42 (95% CI, 0.08–2.19). Six remission events were reported. Because both estimates were derived from a single trial, between-study heterogeneity was not applicable. Study-specific definitions are provided in Supplementary Table 3, and the corresponding effect estimates are presented in Table 2.

### 3.6 Treatment discontinuation and adverse events

Seven trials involving 403 participants reported extractable data on all-cause discontinuation. The pooled risk ratio was 1.15 (95% CI, 0.67–1.98), with I^2^ = 1.9%, *Q*-test *P* = 0.410, and τ^2^ = 0. The 95% prediction interval was 0.67–1.98.

Discontinuation due to adverse events was available from one trial involving 50 participants. One event was reported, and the risk ratio calculated with a continuity correction was 0.33 (95% CI, 0.01–7.81).

Five reports involving 368 participants provided information on overall adverse events. These data were not quantitatively pooled because event definitions or arm-specific event counts were incomplete.

Gastrointestinal adverse-event data were available from one trial involving 60 participants. Three events were reported, and the risk ratio was 0.50 (95% CI, 0.05–5.22).

Four trials involving 294 participants explicitly reported serious adverse-event data. No serious adverse events were observed, and a risk ratio could not be estimated.

### 3.7 Subgroup analyses

Subgroup analyses were conducted according to probiotic formulation, daily probiotic dose, intervention duration, treatment context, diagnostic population, and outcome rater. All interaction *P*-values were greater than 0.05.

For probiotic formulation, the pooled standardized mean difference was −0.36 (95% CI, −1.80 to 1.09) in three single-strain trials and −0.44 (95% CI, −0.81 to −0.07) in six multistrain trials. The *P*-value for interaction was 0.817.

For daily probiotic dose, the pooled standardized mean differences were −0.49 (95% CI, −1.15 to 0.17) for doses below 1 × 10^10^ CFU/day, −0.42 (95% CI, −1.18 to 0.33) for doses from 1 × 10^10^ to below 1 × 10^11^ CFU/day, and −0.19 (95% CI, −0.79 to 0.42) for doses of at least 1 × 10^11^ CFU/day. The *P*-value for interaction was 0.819.

For intervention duration, the pooled standardized mean difference was −0.43 (95% CI, −3.46 to 2.60) in two trials lasting 31 days or less and −0.43 (95% CI, −0.83 to −0.03) in seven trials lasting more than 31 days. The *P*-value for interaction was 0.993.

For treatment context, the pooled standardized mean difference was −0.49 (95% CI, −0.85 to −0.13) in seven adjunctive-treatment trials and −0.28 (95% CI, −4.67 to 4.11) in two trials with a mixed or unclear treatment context. The *P*-value for interaction was 0.438.

For diagnostic population, the pooled standardized mean difference was −0.56 (95% CI, −0.89 to −0.23) in seven trials restricted to major depressive disorder and −0.04 (95% CI, −1.32 to 1.24) in two trials enrolling heterogeneous depressive-disorder populations. The *P*-value for interaction was 0.100.

For outcome rater, the pooled standardized mean difference was −0.37 (95% CI, −0.77 to 0.03) in seven trials using clinician-rated measures and −0.64 (95% CI, −1.61 to 0.33) in two trials using self-reported measures. The *P*-value for interaction was 0.387. Full subgroup results are presented in Table 3.

**TABLE 3:** Subgroup analyses of probiotic effects on depressive symptom severity.

Subgroup variable	Subgroup category	No. of studies	Sample size	SMD	95% CI	Within-subgroup I^2^ (%)	Within-subgroup heterogeneity *P*-value	P for subgroup difference/ interaction
Formulation	Single strain	3	137	−0.36	−1.80 to 1.09	68.60	0.041	0.817
Formulation	Multistrain	6	323	−0.44	−0.81 to −0.07	42.20	0.124	–
Daily probiotic dose	<1 × 10^10^ CFU/day	4	227	−0.49	−1.15 to 0.17	61.60	0.050	0.819
Daily probiotic dose	1 × 10^10^ to <1 × 10^11^ CFU/day	4	190	−0.42	−1.18 to 0.33	54.50	0.086	–
Daily probiotic dose	≥1 × 10^11^ CFU/day	1	43	−0.19	−0.79 to 0.42	0.00	Not applicable	–
Duration	≤31 days	2	88	−0.43	−3.46 to 2.60	16.40	0.274	0.993
Duration	>31 days	7	372	−0.43	−0.83 to −0.03	56.60	0.032	–
Treatment context	Adjunctive	7	321	−0.49	−0.85 to −0.13	35.10	0.161	0.438
Treatment context	Mixed/unclear	2	139	−0.28	−4.67 to 4.11	71.40	0.061	–
Diagnostic population	MDD only	7	323	−0.56	−0.89 to −0.23	26.80	0.224	0.100
Diagnostic population	Heterogeneous depressive disorders	2	137	−0.04	−1.32 to 1.24	0.00	0.557	–
Outcome rater	Clinician-rated	7	367	−0.37	−0.77 to 0.03	55.20	0.037	0.387
Outcome rater	Self-reported	2	93	−0.64	−1.61 to 0.33	0.00	0.721	–

Kazemi et al. reported the probiotic dose only as ≥1 × 10^10^ CFU/day and was classified in the 1 × 10^10^ to <1 × 10^11^ CFU/day subgroup for the present analysis. All subgroup study counts, sample sizes, effect estimates, heterogeneity statistics, and interaction *P*-values are unchanged.

### 3.8 Leave-one-out sensitivity analysis

Leave-one-out sensitivity analysis was performed for depressive symptom severity by sequentially excluding each of the nine trials. The re-estimated pooled standardized mean differences ranged from −0.52 to −0.38. The corresponding lower and upper limits of the 95% confidence intervals ranged from −0.82 to −0.05. All re-estimated standardized mean differences remained negative, and all 95% confidence intervals excluded zero.

The I^2^ values ranged from 26.3% to 52.1%, and τ^2^ ranged from 0.016 to 0.087. The complete leave-one-out results are presented in Supplementary Table 5.

### 3.9 Assessment of small-study effects

Formal assessment of funnel-plot asymmetry and regression-based small-study effects was not used for inferential conclusions because nine studies were available for the primary outcome.

### 3.10 Certainty of evidence

Using the GRADE framework, the certainty of evidence was rated as moderate for depressive symptom severity, clinical response, remission, gastrointestinal adverse events, and serious adverse events. The certainty of evidence for all-cause discontinuation was rated as high. The detailed domain-level judgments and downgrading rationales are provided in Supplementary Table 6.

## 4 Discussion

This systematic review and meta-analysis provides an updated synthesis of randomized evidence on probiotic supplementation in adults with clinically established depressive disorders. A notable contribution is the restriction to clinically diagnosed populations rather than samples defined solely by elevated depressive symptom scores, together with detailed characterization of diagnostic criteria, treatment context, probiotic strains, daily dose, and intervention duration (29). The review also evaluated not only depressive symptom severity but also clinical response, remission, treatment discontinuation, and adverse events, thereby distinguishing symptomatic improvement from broader indicators of clinical benefit and treatment acceptability. Across nine trials involving 460 participants, probiotic supplementation was associated with a modest reduction in depressive symptom severity, with a pooled Hedges’ g of −0.43. The use of restricted maximum likelihood estimation, Hartung–Knapp confidence intervals, prediction intervals, leave-one-out analyses, and GRADE assessment strengthened the interpretation of the pooled evidence. Clinically, these findings suggest that probiotics may have value as an adjunctive option within comprehensive depression care, particularly because most included trials evaluated supplementation alongside antidepressant therapy or treatment as usual. However, the observed effect represents a standardized average across different depression scales rather than a directly transferable change in HAMD, MADRS, or BDI scores. Accordingly, the results support cautious consideration of probiotics as a complementary strategy while preserving established pharmacotherapy and psychotherapy as the foundation of treatment (30).

Beyond the pooled average effect, the heterogeneity and sensitivity analyses provide important information regarding the consistency and potential applicability of the finding. Between-study heterogeneity was moderate (I^2^ = 46.8%; τ^2^ = 0.069), indicating that the magnitude of the difference in depressive symptom severity varied across the included trials (31). Such variation may reflect differences in diagnostic populations, baseline symptom severity, background psychiatric treatment, probiotic composition, daily dose, intervention duration, and outcome assessment. The 95% prediction interval ranged from −1.10 to 0.25 and crossed the null, suggesting that the effect expected in a future comparable setting may range from a relatively pronounced improvement to little or no difference between groups (32). This contrasts with the statistically significant pooled confidence interval and highlights the distinction between the precision of the mean effect and the dispersion of effects across settings (33). Nevertheless, the leave-one-out analysis showed that sequential exclusion of individual studies produced pooled SMDs ranging from −0.52 to −0.38, with all confidence intervals remaining below zero. The overall direction and statistical significance of the pooled estimate were therefore not dependent on any single trial. Considered together, these findings suggest that the average association was reasonably robust within the available evidence base, while its magnitude may vary across clinical and intervention contexts. The pooled result should consequently be interpreted as an overall treatment signal rather than an expectation of uniform benefit for every probiotic formulation, depressive-disorder population, or treatment environment.

The exploratory subgroup analyses did not demonstrate statistically significant effect modification by probiotic formulation, daily dose, intervention duration, treatment context, diagnostic population, or outcome rater, as all interaction tests yielded *P*-values greater than 0.05. Accordingly, statistical significance within an individual subgroup should not be interpreted as evidence that the corresponding subgroup differed from its comparator subgroup (34). For example, the pooled estimate favored probiotics among trials restricted to major depressive disorder, whereas the estimate was close to the null in studies enrolling heterogeneous depressive-disorder populations; however, the interaction test did not confirm a differential effect. Similarly, the findings did not establish superiority of multistrain over single-strain formulations, higher over lower doses, longer over shorter interventions, or clinician-rated over self-reported outcomes. The current subgroup results therefore do not support selection of a specific probiotic formulation, dose range, or treatment duration on the basis of comparative efficacy. The secondary outcomes also differed from the continuous symptom-severity finding. Clinical response and remission were evaluable from only one trial, and their risk-ratio estimates had wide confidence intervals crossing the null. Thus, the observed reduction in standardized symptom scores was not accompanied by evidence of improved categorical response or remission. All-cause discontinuation likewise showed no clear between-group difference (RR, 1.15; 95% CI, 0.67–1.98), suggesting broadly similar treatment continuation across groups. Discontinuation due to adverse events and gastrointestinal adverse events were based on very few events, while overall adverse events could not be pooled because reporting definitions and arm-specific counts were incomplete. No serious adverse events were observed in the trials that explicitly reported this outcome, although the absence of observed events does not establish the absence of rare harms (35). These findings distinguish a possible symptomatic effect from still-uncertain effects on clinically defined improvement, acceptability, and safety.

Recent meta-analyses have generally identified a modest beneficial association between probiotic supplementation and depressive symptom severity, although their populations and analytical approaches differed. Tam reported a pooled standardized mean difference of 0.38 favoring probiotics across 13 studies involving 710 patients with major depressive disorder, with moderate heterogeneity (I^2^ = 42.8%) and no significant moderators identified by subgroup analysis or meta-regression. Liu et al. (36) similarly found a small reduction in depressive symptoms among unmedicated adults (six randomized controlled trials; SMD, −0.38; 95% CI, −0.57 to −0.18; I^2^ = 51%); however, the estimate became nonsignificant after industry-funded studies or trials involving adjunctive agents were excluded, suggesting that the magnitude of benefit may depend on study context. Shakir et al. (37) reported significant improvement in Hamilton Depression Rating Scale scores across six studies involving 378 participants, with comparable heterogeneity (I^2^ = 45%), but found no significant effects on interleukin-6 or tumor necrosis factor-α, indicating that symptomatic improvement was not accompanied by consistent changes in the evaluated inflammatory markers. Zhao et al. (38) reported a larger effect of probiotics versus placebo in a network meta-analysis (SMD, −0.62; 95% credible interval, −0.86 to −0.42), although this estimate incorporated indirect comparisons across 22 interventions and evidence certainty ranged from moderate to very low. In contrast, Rahmannia et al. (39) found significant improvement on the Beck Depression Inventory but not on the Hamilton Depression Rating Scale, Depression Anxiety Stress Scales, or Montgomery–Åsberg Depression Rating Scale, highlighting the potential influence of outcome measurement on pooled conclusions. Our estimate (SMD, −0.43; 95% CI, −0.73 to −0.12) was broadly consistent with the small-to-moderate effects reported previously, but was derived exclusively from adults with clinically established depressive disorders. The prediction interval crossed the null, and evidence for clinical response, remission, and safety remained inconclusive, supporting a cautious interpretation of probiotics as a possible adjunct rather than an established substitute for conventional depression treatment.

### 4.1 Limitations and future research

Several limitations should be considered. The evidence base was limited by the small number and sample sizes of the included trials, which reduced the precision of pooled estimates, limited the reliability of exploratory subgroup analyses, and restricted the detection of uncommon adverse events. Clinical and methodological heterogeneity remained across patient populations, treatment settings, concomitant therapies, probiotic formulations, doses, intervention durations, and outcome measures. Although standardized mean differences enabled synthesis across different depression scales, the pooled effect could not be directly translated into a uniform scale-specific change or a clearly defined patient-level clinical benefit. Evidence for response, remission, and safety was also limited, and follow-up was generally insufficient to evaluate sustained efficacy, relapse prevention, and long-term safety. In addition, publication bias could not be assessed reliably because of the limited number of studies. Future trials should include larger and well-characterized populations, standardized diagnostic and outcome definitions, prespecified clinically meaningful endpoints, longer follow-up, and comprehensive reporting of probiotic strains, doses, adherence, and adverse events. Prospective registration and complete outcome reporting are also needed to clarify which patients and probiotic strategies may derive clinically relevant benefit.

## 5 Conclusion

In summary, probiotic supplementation may modestly reduce depressive symptom severity in adults with clinically diagnosed depressive disorders. However, the prediction interval included the null, and evidence for clinical response, remission, and safety remained inconclusive. Probiotics may have potential as an adjunctive intervention but should not replace established pharmacotherapy or psychotherapy. Interpretation is limited by small sample sizes, short treatment durations, variation in probiotic formulations and doses, heterogeneous populations and outcome measures, and incomplete safety reporting. Larger, adequately powered randomized controlled trials with standardized diagnostic criteria, prespecified clinically meaningful outcomes, longer follow-up, and detailed strain- and dose-specific reporting are needed.

## Data Availability

The raw data supporting the conclusions of this article will be made available by the authors, without undue reservation.
