# Supplementary material for: Clinical effects of probiotic supplementation on depressive symptom severity in adults with depressive disorders: a systematic review and meta-analysis
Source: Front Nutr. 2026 Oct 5;13:1900466. doi: 10.3389/fnut.2026.1900466 (PMC13677502; doi:10.3389/fnut.2026.1900466)
Supplement: Supplementary file 1 [file Table_1.docx]

Supplementary Table S1. Search Strategy for Four Databases

| Database | Search Strategy |
| --- | --- |
| PubMed | (“Probiotics”[Mesh] OR “Lactobacillus”[Mesh] OR “Bifidobacterium”[Mesh] OR probiotic*[Title/Abstract] OR psychobiotic*[Title/Abstract] OR Lactobacillus[Title/Abstract] OR Bifidobacterium[Title/Abstract] OR Bacillus[Title/Abstract] OR “Clostridium butyricum”[Title/Abstract] OR “gut microbiota”[Title/Abstract] OR microbiome[Title/Abstract]) AND (“Depressive Disorder”[Mesh] OR “Depression”[Mesh] OR depression[Title/Abstract] OR depressive[Title/Abstract] OR “depressive disorder”[Title/Abstract] OR “major depressive disorder”[Title/Abstract] OR “major depression”[Title/Abstract] OR MDD[Title/Abstract] OR “depressive symptoms”[Title/Abstract]) AND (“Randomized Controlled Trial”[Publication Type] OR randomized[Title/Abstract] OR randomised[Title/Abstract] OR placebo[Title/Abstract] OR “placebo-controlled”[Title/Abstract] OR trial[Title/Abstract] OR “clinical trial”[Title/Abstract] OR “double-blind”[Title/Abstract]) |
| Embase | (‘probiotic agent’/exp OR ‘lactobacillus’/exp OR ‘bifidobacterium’/exp OR probiotic*:ti,ab,kw OR psychobiotic*:ti,ab,kw OR lactobacillus:ti,ab,kw OR bifidobacterium:ti,ab,kw OR bacillus:ti,ab,kw OR ‘clostridium butyricum’:ti,ab,kw OR ‘gut microbiota’:ti,ab,kw OR microbiome:ti,ab,kw) AND (‘depression’/exp OR ‘depressive disorder’/exp OR depression:ti,ab,kw OR depressive:ti,ab,kw OR ‘depressive disorder’:ti,ab,kw OR ‘major depressive disorder’:ti,ab,kw OR ‘major depression’:ti,ab,kw OR mdd:ti,ab,kw OR ‘depressive symptoms’:ti,ab,kw) AND (‘randomized controlled trial’/exp OR randomized:ti,ab,kw OR randomised:ti,ab,kw OR random*:ti,ab,kw OR placebo:ti,ab,kw OR ‘placebo controlled’:ti,ab,kw OR trial:ti,ab,kw OR ‘clinical trial’:ti,ab,kw OR ‘double blind’:ti,ab,kw) |
| Web of Science | TS=(probiotic* OR psychobiotic* OR Lactobacillus OR Bifidobacterium OR Bacillus OR “Clostridium butyricum” OR “gut microbiota” OR microbiome) AND TS=(depression OR depressive OR “depressive disorder” OR “major depressive disorder” OR “major depression” OR MDD OR “depressive symptoms”) AND TS=(random* OR randomized OR randomised OR placebo OR “placebo-controlled” OR trial OR “clinical trial” OR “randomized controlled trial” OR “double blind”) |
| Cochrane Library | (probiotic* OR psychobiotic* OR Lactobacillus OR Bifidobacterium OR Bacillus OR “Clostridium butyricum” OR “gut microbiota” OR microbiome):ti,ab,kw AND (depression OR depressive OR “depressive disorder” OR “major depressive disorder” OR “major depression” OR MDD OR “depressive symptoms”):ti,ab,kw AND (randomized OR randomised OR random* OR placebo OR “placebo-controlled” OR trial OR “clinical trial” OR “randomized controlled trial” OR “double blind”):ti,ab,kw |

Supplementary Table S2. Detailed probiotic strains, doses, durations, and comparators

| Study | Product name | Manufacturer | All genera, species, and strain identifiers | Single-/multistrain | CFU per strain | Total CFU/dose | Administration frequency | Total CFU/day | Dosage form | Duration | Background treatment | Comparator type | Placebo composition | Placebo matching | Adherence method | Adherence result |
| --- | --- | --- | --- | --- | --- | --- | --- | --- | --- | --- | --- | --- | --- | --- | --- | --- |
| Elahinejad et al., 2025 | R0052/R0175 probiotic capsule | Takgen Pharma, Tehran, Iran | Lactobacillus helveticus R0052 + Bifidobacterium longum R0175 | Multistrain (2 strains) | 3×10⁹ CFU/day per strain | 6×10⁹ CFU/capsule | Once daily | 6×10⁹ CFU/day | Capsule | 8 weeks | Fluoxetine initiated concurrently | Placebo | Starch | Identical appearance and packaging | Returned capsule count plus daily diary; adherence threshold ≥80% | 90% probiotic versus 88% placebo |
| Lin et al., 2024 | PS128 capsule | Bened Biomedical Co., Ltd. | Lactiplantibacillus plantarum PS128; DSM 28632 | Single strain | 3×10¹⁰ CFU/capsule | 3×10¹⁰ CFU/capsule | Twice daily | 6×10¹⁰ CFU/day | Capsule | 8 weeks | Stable antidepressant and other psychotropic treatment for at least 1 month | Placebo | Microcrystalline cellulose | Visually identical capsules | Returned unused capsules | Dose adherence percentage not reported; all 32 participants completed the week-8 assessment |
| Gawlik-Kotelnicka et al., 2024 | Sanprobi Stress | Sanprobi; probiotic powder supplied by Institute Rosell-Lallemand | Lactobacillus helveticus Rosell-52/R0052 + Bifidobacterium longum Rosell-175/R0175 | Multistrain (2 strains) | Individual strain allocation not specified | 3×10⁹ CFU/capsule | Once daily | 3×10⁹ CFU/day | Capsule | 60 days | Usual psychiatric care; antidepressants and some antipsychotic treatment | Placebo | Capsule containing excipients only | Same capsule and coded packaging | Daily medication log | All included completers were reported as compliant |
| Nikolova et al., 2023 | Bio-Kult Advanced | ADM Protexin Ltd. | Bacillus subtilis PXN 21; Bifidobacterium bifidum PXN 23; B. breve PXN 25; B. infantis PXN 27; B. longum PXN 30; Lactobacillus acidophilus PXN 35; L. delbrueckii subsp. bulgaricus PXN 39; L. casei PXN 37; L. helveticus PXN 45; L. plantarum PXN 47; L. rhamnosus PXN 54; L. salivarius PXN 57; Lactococcus lactis subsp. lactis PXN 63; Streptococcus thermophilus PXN 66 | Multistrain (14 strains) | Individual strain allocation not disclosed | 2×10⁹ CFU/capsule | 4 capsules daily | 8×10⁹ CFU/day | Capsule | 8 weeks | Stable approved antidepressant treatment for at least 6 weeks | Placebo | Microcrystalline cellulose in a hydroxypropyl-methylcellulose capsule | Visually identical capsules and identical packaging | Returned capsule count and study visits | Mean adherence 97.2% |
| Tian et al., 2022 | Bifidobacterium breve CCFM1025 | Shisheng Yisheng (Yangzhou) Biotechnology Co., Ltd. | Bifidobacterium breve CCFM1025 | Single strain | 1×10¹⁰ CFU/sachet | 1×10¹⁰ CFU/sachet | Once daily | 1×10¹⁰ CFU/day | Freeze-dried powder in sachet | 4 weeks | Usual antidepressant treatment; agents and regimens were not standardized | Placebo | Maltodextrin | Matched sachets and blinded coding | Dose-adherence assessment method not reported | Dose adherence percentage not reported; trial completion was 80.0% in the probiotic group and 96.2% in the placebo group |
| Schaub et al., 2022 | Vivomixx | Mendes SA | Streptococcus thermophilus; Bifidobacterium breve; B. longum/B. lactis; B. infantis/B. lactis; Lactobacillus acidophilus; L. plantarum; L. paracasei; L. delbrueckii subsp. bulgaricus/L. helveticus; NCIMB 30438, 30441, 30435, 30436, 30442, 30437, 30439, and 30440 | Multistrain (8 strains) | Individual strain allocation not specified | 9×10¹¹ CFU/sachet | Once daily | 9×10¹¹ CFU/day | Sachet mixed into a cold drink | 31 days | Inpatient treatment as usual | Placebo | Maltose | Identical color, shape, size, packaging, smell, and taste | Returned sachets and clinical administration records; mITT adherence threshold >65% | Mean adherence approximately 88% |
| Rudzki et al., 2019 | Sanprobi IBS | Sanprobi; strain/capsule material supplied by Institute Rosell-Lallemand | Lactiplantibacillus plantarum 299v / DSM 9843 | Single strain | 1×10¹⁰ CFU/capsule | 1×10¹⁰ CFU/capsule | Twice daily | 2×10¹⁰ CFU/day | Capsule | 8 weeks | SSRI treatment | Placebo | Crystalline cellulose | Matched capsules under double-blind coding | Empty blister return every 4 weeks | Dose adherence percentage not reported; trial completion was 75.0% in the probiotic group and 76.9% in the placebo group |
| Kazemi et al., 2019 | CEREBIOME | Lallemand Health Solutions | Lactobacillus helveticus R0052/CNCM I-1722 + Bifidobacterium longum R0175/CNCM I-3470 | Multistrain (2 strains) | Individual strain allocation not specified | ≥1×10¹⁰ CFU per 5-g sachet | Once daily | ≥1×10¹⁰ CFU/day | Orally dispersible 5-g sachet | 8 weeks | Stable antidepressant medication | Placebo | Xylitol, maltodextrin, malic acid, and plum flavor | Same appearance and taste | Returned sachet count; adherence threshold ≥80% | 91.9%±5.53% |
| Akkasheh et al., 2016 | Three-strain probiotic capsule | Tak Gen Zist Pharmaceutical Co. | Lactobacillus acidophilus + Lactobacillus casei + Bifidobacterium bifidum; strain identifiers not reported | Multistrain (3 species) | 2×10⁹ CFU/day per species | 6×10⁹ CFU/capsule | Once daily | 6×10⁹ CFU/day | Capsule | 8 weeks | Adjunctive to citalopram | Placebo | Starch | Indistinguishable capsules | Returned containers and capsule count | >90% |

Supplementary Table S3. Diagnostic criteria and clinical outcome definitions

| Study | Depressive disorder | DSM/ICD criteria | Structured diagnostic tool | Diagnostic assessor | Baseline severity threshold | Meta-analysis scale and version | Endpoint or change | Assessment time | Clinical response definition | Remission definition | Analysis population | IBS status | Other key comorbidity restrictions |
| --- | --- | --- | --- | --- | --- | --- | --- | --- | --- | --- | --- | --- | --- |
| Elahinejad et al., 2025 | First-episode MDD | DSM-IV-TR | Clinical psychiatric interview; instrument not named | Psychiatrist/clinical assessor | HDRS-24 ≥15; no antidepressant use for ≥2 months | HDRS-24 | Endpoint median/IQR; SMD calculated | Week 8 | Not assessed/reported | Not assessed/reported | PP completers: 23 versus 21 | IBS was not an eligibility requirement | Excluded major medical disease, pregnancy/lactation, psychosis/mania, substance abuse, and recent probiotics, antibiotics, supplements, or psychotherapy |
| Lin et al., 2024 | MDD | DSM-5 | Psychiatrist-confirmed DSM-5 major depressive episode | Psychiatrist | Moderate symptom severity; exact HAMD-17 threshold not stated | HAMD-17 | Published study-level SMD | Week 8 | Not assessed/reported | Not assessed/reported | 32 participants; 16 per group | IBS was not an eligibility requirement | Han Chinese adults aged 20–65 years; stable antidepressant and psychotropic treatment; complete exclusion details were not available in the accessible report |
| Gawlik-Kotelnicka et al., 2024 | Depressive episode, recurrent depressive disorder, or mixed depressive-anxiety disorder | ICD-11 codes 6A70, 6A71, and 6A73 | Clinical psychiatric diagnosis | Clinician | MADRS ≥13 | MADRS | Endpoint | Day 60 | ≥50% reduction from baseline | MADRS <5 | PP; final MADRS analysis: 51 versus 43 | IBS was not an eligibility requirement | Metabolic syndrome and abdominal obesity were prespecified stratification/effect-modification variables |
| Nikolova et al., 2023 | MDD with incomplete response to antidepressant treatment | DSM-5 | Clinical diagnostic assessment; instrument not named | Trained blinded clinician | HAMD-17 >13; stable approved antidepressant treatment for ≥6 weeks | HAMD-17 | Endpoint | Week 8 | Not assessed/reported | Not assessed/reported | ITT/mixed-model population: 24 versus 25 | IBS was not an eligibility requirement | Age 18–55; excluded bipolar disorder, psychosis, eating disorder, recent substance dependence, active suicidal ideation, significant gastrointestinal disease/surgery, recent antibiotics, and regular probiotics |
| Tian et al., 2022 | MDD | Clinical diagnosis; formal diagnostic criteria not fully detailed | Clinical assessment; instrument not named | Clinical doctors/assessors | Intended HDRS-24 >14; protocol deviations were reported | HDRS-24 | Endpoint | Week 4 | Not assessed/reported | Not assessed/reported | PP completers: 20 versus 25 | IBS was not an eligibility requirement | Excluded severe/refractory or suicidal illness, serious physical/neurological disease, and pregnancy/lactation |
| Schaub et al., 2022 | Current depressive episode across affective disorders | ICD-10 categories F31.3–F34 | Clinical diagnosis; instrument not named | Trained clinical raters | HAMD >7 after protocol relaxation | HAMD; item count not stated | Published study-level SMD and longitudinal estimate | End of 31-day intervention | >57% reduction from baseline | Not reported | ITT models; SMD population: 19 versus 24 | IBS was not an eligibility requirement | Hospitalized affective-disorder population; detailed psychiatric and medical exclusions applied |
| Rudzki et al., 2019 | MDD | DSM-IV | Clinical psychiatric interview; instrument not named | Psychiatrist | Eligibility threshold not stated; baseline HAMD-17 was 21.53±6.03 versus 22.00±7.92 | HAMD-17 | Published study-level SMD | Week 8 | Not assessed/reported | Not assessed/reported | PP completers: 30 versus 30 | Sanprobi IBS was the product name; participants were not selected for IBS | Excluded inflammatory, oncologic, autoimmune, metabolic, neurologic and other psychiatric disorders; smoking, pregnancy/lactation, BMI <18.5 or >30, substance misuse, and selected medications |
| Kazemi et al., 2019 | Mild-to-moderate MDD with melancholic features | DSM-IV-based clinical diagnosis | Clinical psychiatric assessment | Psychiatrist; BDI-II self-report | Current mild-to-moderate depression for ≥1 month | BDI-II | Endpoint | Week 8 | Not assessed/reported | Not assessed/reported | PP dataset: 28 versus 25 | IBS was not an eligibility requirement | Adults aged 20–50 years with protocol-specified medical, dietary, pregnancy, medication, antibiotic, and supplement exclusions |
| Akkasheh et al., 2016 | MDD | DSM-IV | Clinical psychiatric diagnosis; HAMD-17 used for eligibility | Psychiatrist; BDI self-report | HAMD-17 ≥15; age 20–55 years | BDI, 21-item; version not stated | Change from baseline | Week 8 | Not assessed/reported | Not assessed/reported | ITT with last observation carried forward: 20 versus 20; completers 17 versus 18 | IBS was not an eligibility requirement | Excluded coronary infarction/angina, pregnancy/lactation, substance misuse, and recent probiotic or dietary supplements |

Supplementary Table S4. Outcome selection and data-processing decisions

| Study | Outcome domain | Alternative scales reported | Final selected scale | Selection rationale | Selected time point | Endpoint/change | Analysis population | N probiotic | N control | Probiotic value | Probiotic SD/IQR | Control value | Control SD/IQR | Data source | Data processed? | Processing method | Multi-arm handling |
| --- | --- | --- | --- | --- | --- | --- | --- | --- | --- | --- | --- | --- | --- | --- | --- | --- | --- |
| Elahinejad et al., 2025 | Depressive symptom severity | HDRS-24 | HDRS-24 | Only reported depression scale | Week 8 | Endpoint median/IQR | PP | 23 | 21 | 7 | IQR 4–10 | 11 | IQR 8–19 | Included article | Yes | Mean=(Q1+median+Q3)/3; SD=IQR/1.35 | Not applicable |
| Lin et al., 2024 | Depressive symptom severity | HAMD-17; DSSS | HAMD-17 | Clinician-rated depression scale prioritized | Week 8 | Published study-level SMD | 32 participants; 16 per group | 16 | 16 | 0.36 | 95% CI −0.33 to +1.06 |  |  | Tam et al. 2026 | Yes | Direction aligned so negative values favor probiotics | Not applicable |
| Gawlik-Kotelnicka et al., 2024 | Depressive symptom severity | MADRS; DASS | MADRS | Primary clinician-rated depression outcome | Day 60 | Endpoint | PP | 51 | 43 | 16.1 | 6.4 | 15.9 | 7.8 | Included article | Yes | Hedges g calculated from endpoint means and SDs | Not applicable |
| Nikolova et al., 2023 | Depressive symptom severity | HAMD-17; IDS-SR | HAMD-17 | Clinician-rated scale prioritized | Week 8 | Endpoint | ITT/mixed-model population | 24 | 25 | 8.83 | 4.28 | 11.09 | 3.22 | Included article | Yes | Hedges g calculated from endpoint means and SDs | Not applicable |
| Tian et al., 2022 | Depressive symptom severity | HDRS-24; MADRS; BPRS | HDRS-24 | Principal clinician-rated scale; endpoint selected because the reported change score was inconsistent with baseline and endpoint values | Week 4 | Endpoint | PP | 20 | 25 | 8.9 | 6.4 | 13.92 | 8.09 | Included article | Yes | Hedges g calculated from endpoint means and SDs | Not applicable |
| Schaub et al., 2022 | Depressive symptom severity | HAMD; BDI | HAMD | Clinician-rated primary outcome | End of 31-day intervention | Published study-level SMD | mITT | 19 | 24 | −0.19 | 95% CI −0.79 to +0.42 |  |  | Tam et al. 2026 and included article | Yes | Direction aligned so negative values favor probiotics | Not applicable |
| Rudzki et al., 2019 | Depressive symptom severity | HAMD-17; SCL-90; PSS-10 | HAMD-17 | Clinician-rated depression scale prioritized | Week 8 | Published study-level SMD | PP | 30 | 30 | −0.67 | 95% CI −1.19 to −0.15 |  |  | Tam et al. 2026 and included article | Yes | Direction aligned so negative values favor probiotics | Not applicable |
| Kazemi et al., 2019 | Depressive symptom severity | BDI-II | BDI-II | Only depression scale; numerical endpoint supplied by a related report | Week 8 | Endpoint | PP dataset | 28 | 25 | 9.8 | 8 | 15.9 | 12.7 | Related report from the same trial | Yes | Hedges g calculated from endpoint means and SDs | Prebiotic arm excluded; placebo used once |
| Akkasheh et al., 2016 | Depressive symptom severity | BDI | BDI | Only extractable depression outcome | Week 8 | Change from baseline | ITT with last observation carried forward | 20 | 20 | −5.7 | 6.4 | −1.5 | 4.8 | Included article | Yes | Hedges g calculated from change-score means and SDs | Not applicable |

Supplementary Table S5. Leave-one-out sensitivity analyses

| Outcome | Excluded study | Remaining studies | Remaining sample size | Repooled SMD | 95% CI | I² (%) | τ² | Effect direction maintained? | Statistical significance maintained? | Single-study dominance? |
| --- | --- | --- | --- | --- | --- | --- | --- | --- | --- | --- |
| Depressive symptom severity | Elahinejad et al., 2025 | 8 | 416 | −0.38 | −0.70 to −0.05 | 45.1 | 0.063 | Yes | Yes | No; no omission reversed the direction or statistical significance |
| Depressive symptom severity | Lin et al., 2024 | 8 | 428 | −0.49 | −0.76 to −0.23 | 30.1 | 0.042 | Yes | Yes | No; no omission reversed the direction or statistical significance |
| Depressive symptom severity | Gawlik-Kotelnicka et al., 2024 | 8 | 366 | −0.52 | −0.82 to −0.22 | 26.3 | 0.016 | Yes | Yes | No; no omission reversed the direction or statistical significance |
| Depressive symptom severity | Nikolova et al., 2023 | 8 | 411 | −0.41 | −0.76 to −0.06 | 52 | 0.086 | Yes | Yes | No; no omission reversed the direction or statistical significance |
| Depressive symptom severity | Tian et al., 2022 | 8 | 415 | −0.40 | −0.74 to −0.06 | 50.8 | 0.081 | Yes | Yes | No; no omission reversed the direction or statistical significance |
| Depressive symptom severity | Schaub et al., 2022 | 8 | 417 | −0.46 | −0.80 to −0.11 | 51.7 | 0.082 | Yes | Yes | No; no omission reversed the direction or statistical significance |
| Depressive symptom severity | Rudzki et al., 2019 | 8 | 400 | −0.39 | −0.74 to −0.05 | 49.6 | 0.08 | Yes | Yes | No; no omission reversed the direction or statistical significance |
| Depressive symptom severity | Kazemi et al., 2019 | 8 | 407 | −0.41 | −0.76 to −0.06 | 52.1 | 0.087 | Yes | Yes | No; no omission reversed the direction or statistical significance |
| Depressive symptom severity | Akkasheh et al., 2016 | 8 | 420 | −0.39 | −0.73 to −0.06 | 49.9 | 0.076 | Yes | Yes | No; no omission reversed the direction or statistical significance |

Supplementary Table S6. GRADE evidence profile

| Outcome | No. of studies | Total sample size | Pooled effect (95% CI) | Risk of bias | Inconsistency | Indirectness | Imprecision | Publication bias | Final certainty of evidence | Downgrading rationale |
| --- | --- | --- | --- | --- | --- | --- | --- | --- | --- | --- |
| Depressive symptom severity | 9 | 460 | SMD −0.43 (−0.73 to −0.12) | Not serious | Not serious | Not serious | Serious | Not serious | Moderate | Downgraded one level for imprecision because of the limited sample size and a wide confidence interval. |
| Clinical response | 1 | 94 | RR 0.75 (0.32 to 1.77) | Not serious | Not applicable | Serious | Not serious | Cannot be assessed | Moderate | Downgraded one level for indirectness because the study population, intervention, or outcome did not fully match the review question. |
| Remission | 1 | 94 | RR 0.42 (0.08 to 2.19) | Not serious | Serious | Not serious | Not serious | Not serious | Moderate | Downgraded one level for inconsistency because of substantial heterogeneity or variation in effect estimates across studies. |
| All-cause discontinuation | 7 | 403 | RR 1.15 (0.67 to 1.98) | Not serious | Not serious | Not serious | Not serious | Not serious | High | No downgrading was required because no serious concerns were identified across the GRADE domains. |
| Gastrointestinal adverse events | 1 | 60 | RR 0.50 (0.05 to 5.22) | Serious | Not applicable | Not serious | Not serious | Cannot be assessed | Moderate | Downgraded one level for risk of bias because of methodological limitations in the included study. |
| Serious adverse events | 4 | 294 | No events; RR not estimable | Not serious | Not applicable | Not serious | Serious | Cannot be assessed | Moderate | Downgraded one level for imprecision because the evidence was based on a small sample or few events, resulting in an uncertain effect estimate. |

Abbreviations: CI, confidence interval; GRADE, Grading of Recommendations Assessment, Development and Evaluation; RR, risk ratio; SMD, standardized mean difference.
